# Effect of Vanillin and Chitin Particles on the Chitosan-Based Oleogels Produced by the Emulsion-Templated Method

**DOI:** 10.3390/gels11100799

**Published:** 2025-10-03

**Authors:** Leticia Montes, Sofía Viciana, Daniel Franco, Jorge Sineiro, Ramón Moreira

**Affiliations:** Department of Chemical Engineering, Universidade de Santiago de Compostela, rúa Lope Gómez de Marzoa, s/n., 15782 Santiago de Compostela, Spain; leticia.montes.martinez@usc.es (L.M.); sofia.viciana.mosquera@usc.es (S.V.); daniel.franco.ruiz@usc.es (D.F.); jorge.sineiro@usc.es (J.S.)

**Keywords:** oil binding capacity, oxidative stability, rheology, storage, texture

## Abstract

This study presents the first assessment of the combined effect of vanillin and chitin particles on the rheological, oil retention, textural, and oxidative properties of chitosan-based oleogels formulated with olive oil. Oleogels were prepared with and without vanillin; in the latter case, the vanillin-to-chitosan ratio was kept constant (1.3), while chitin concentrations (% *w*/*w*) were variable (0.0, 0.5, 1.5, and 2.0). Fresh oleogels and those stored for 15 days were characterized. Results demonstrated that vanillin promotes the formation of compact viscoelastic networks, enhances the elastic modulus by approximately 1.3 times, improves oil binding capacity from 75.1% to 89.2%, and significantly improves oxidative stability by minimizing lipid degradation. In contrast, the influence of chitin was dependent on its content and the presence of vanillin. At intermediate content, chitin positively affected cohesiveness and elasticity, particularly in vanillin-free systems. However, in formulations containing vanillin, even low chitin concentration disrupted the gel network, leading to a decrease in hardness, low oil retention, and a higher oxidation degree. Significant correlations between hardness and elastic modulus, oil binding capacity, adhesiveness, and damping factor were obtained for fresh and stored oleogels.

## 1. Introduction

Excessive consumption of saturated fats has been closely linked to an increased risk of cardiovascular diseases, prompting the food industry to seek healthier alternatives. In this context, oleogels (structured systems based on liquid oils such as olive oil) have emerged as a promising strategy to replace conventional solid fats while maintaining desirable textural and functional properties in food products. These products can mimic the sensory characteristics of saturated fats while providing a healthier lipid profile, rich in monounsaturated fatty acids [1]. Olive oil stands out due to its high content of unsaturated fats and bioactive compounds, offering added nutritional value compared to traditional solid fats. Extra virgin olive oil is rich in polyphenols, tocopherols, and other antioxidants that contribute to its anti-inflammatory and cardioprotective effects. Moreover, its stability under moderate processing conditions and pleasant sensory attributes make it an ideal candidate for use in oleogel-based food formulations. The use of olive oil in oleogels not only improves the nutritional quality of food products but also aligns with current consumer trends favoring clean-label and health-oriented ingredients [2].

Recent studies have demonstrated the feasibility of using oleogels in various food applications, including bakery products, spreads, and processed meats, with the aim of reducing the saturated fat content without compromising quality [3,4]. Additionally, the novel use of biopolymer-based oleogelators, such as chitosan and polysaccharides, have shown promising improvements in the nutritional profile and functional properties of oleogels. Recent studies have also highlighted the potential of oleogels to enhance oxidative stability and sensory acceptance in complex food matrices, further supporting their application as healthier fat alternatives in the food industry [5].

Different strategies for oleogel formation have been explored, either direct (gelator solved into oil) or indirect, when no gelator is directly solved in oil at high temperatures; several approaches are possible. The latter has received special attention because it allows the use of hydrophilic compounds that are commonly used in the food industry (polysaccharides, proteins, etc.). Among indirect approaches, the emulsion-templated method is widely employed; in this method polymers such as cellulose derivatives or chitosan are often used to stabilize the structure and enhance both nutritional and functional properties [6].

Chitosan (a biopolymer derived from chitin found in crustacean exoskeletons with reported antimicrobial and antioxidant activity) facilitates its use as an emulsifier or structuring agent in the aqueous phase [7,8]. The incorporation of vanillin in oleogels intended to be animal fat substitutes in foods or cosmetic formulations has some limitations because of its characteristic flavor [9,10]. Vanillin can react with the amino groups of chitosan to form Schiff bases, increasing the network rigidity and promoting an enhanced oil retention capacity, and delayed oxidative degradation [11,12]. Therefore, even though Schiff-base formation can reinforce gel structures, it is important to assess the intrinsic structuring ability of chitosan alone [13,14]. On the other hand, chitin particles may act as reinforcing agents in the absence of aldehydes such as vanillin, improving the textural strength and thermal stability of oleogels. However, they might either stabilize the network or reduce its homogeneity, depending on their concentration and interaction with vanillin [15].

Specifically, the structured systems including vanillin showed promising features as fat substitutes in ice cream (also because of its specific flavor), although the incorporation procedure significantly influenced several critical quality attributes such as texture, viscosity, melting behavior, and microstructure [16]. Other studies have shown that chitosan-based oleogels (1% *w*/*w*) crosslinked with vanillin exhibited good mechanical and physical stability, particularly when oil was used as the lipid phase, with a controlled release of free fatty acids during digestion, indicating their potential application in functional foods designed to regulate fat intake [17]. More recently, olive oil-based oleogels structured with chitosan–vanillin networks have demonstrated high oil retention, adjustable mechanical properties, and improved oxidative stability. These results reinforce chitosan’s value as a functional, health-promoting, and adaptable structuring agent for fat replacement in food applications [18].

The inclusion of chitin particles within emulsion–template matrices enhances the mechanical strength and thermal stability of oleogels, broadening their applicability in food products. Based on previous findings where chitin stabilizes Pickering emulsions [19], it is hypothesized that chitin could also modulate the physical structure and functional properties of olive oil-based oleogels prepared by the indirect method.

The effect of chitin particles in olive oil-based oleogels prepared by the indirect method remains poorly understood. Specifically, the influence of chitin concentration on the characteristics of oleogels has not been fully elucidated and remains critical for the development of gels with appropriate functional and mechanical properties for food applications.

Most studies involving either chitin particles or vanillin in oleogels preparation were performed on systems containing vanillin or chitin independently, but no study was reported considering their combined effect on the structural and functional characteristics of chitosan-based oleogels. Addressing this gap is essential to determine whether vanillin and chitin act synergistically or antagonistically to define their potential in food applications.

The aim of this study was to develop and characterize olive oleogels prepared by the indirect method, incorporating chitosan, vanillin, and chitin particles. Key properties such as rheology, texture, color, oil retention, and oxidation stability were analyzed to improve the formulation of healthier products with enhanced structure. These results expand the understanding of oleogels as potential fat replacers in the food industry.

## 2. Results and Discussion

### 2.1. Rheological Characterization

Rheological analyses were performed to study the viscoelastic characteristics of tested oleogels, applied on both fresh and stored samples at room temperature and under ambient air conditions for 15 days.

**Figure 1:**
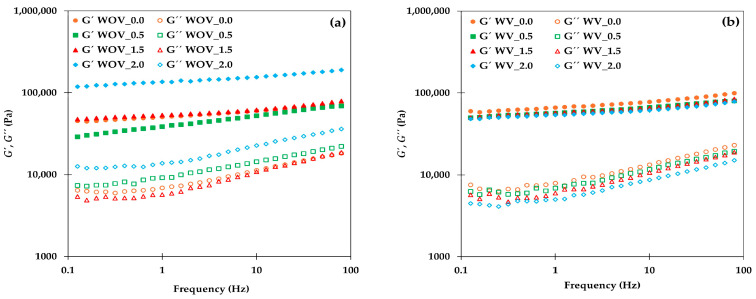

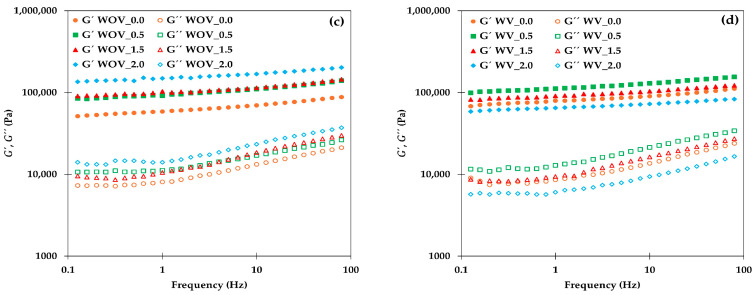
Frequency sweep profiles of chitosan-based oleogels formulated with different chitin content (0, 0.5, 1.5, and 2.0% *w*/*w*): (**a**) fresh oleogels without vanillin (WOV), (**b**) fresh oleogels with vanillin (WV), (**c**) oleogels without vanillin after 15 days, and (**d**) oleogels with vanillin after 15 days.

Strain sweep tests were performed to determine the linear viscoelastic region (LVR), showing all samples a similar behavior: all samples exhibited a plateau up to 10% strain. Consequently, a strain value of 1% was selected for further frequency sweep tests to ensure measurements remained within the LVR. The mechanical spectra of all freshly prepared samples can be seen in Figure 1. In all experiments, the elastic modulus (*G*′) was higher than the viscous modulus (*G*″) throughout the entire frequency range studied, indicating a predominantly elastic (solid-like) behavior. This behavior is consistent with previous findings by Lama et al. [18], who reported similar frequency sweep for chitosan-based oleogels cross-linked with vanillin.

Focusing on the oleogels without chitin, specifically those based on chitosan alone (WOV) and chitosan with vanillin (WV), it was observed that the presence of vanillin increased the elastic modulus at 1 Hz within the tested frequency range, from 50,758 Pa to 66,263 Pa, indicating that the elastic modulus increased by approximately 1.3 times due to the presence of vanillin.

Low chitin content (WOV_0.5 and WOV_1.5) appeared to disrupt the oleogel network in samples without vanillin (Figure 1a), resulting in less structured systems, as indicated by the increased *G*″ for the WOV_0.5 oleogel. However, at a higher concentration (WOV_2.0), a remarkable increase in *G*′ value was observed, suggesting that chitin reinforced the gel structure once a threshold concentration value was reached. This behavior is consistent with findings reported by Gallego et al. [20], who demonstrated that low levels of functionalized chitin incorporated into oleogels led to a decrease in viscoelastic properties, whereas these properties were highly improved above the critical functionalization degree. Although our system does not involve chemical functionalization, these results support the notion that chitin acts as a reinforcing agent in oleogels only when its content is higher than a certain threshold value.

For oleogels containing vanillin (Figure 1b), the addition of chitin seems to have an adverse effect on the mechanical properties, as a general decrease in both viscoelastic moduli was observed. This may be explained by the fact that the chitosan–vanillin system forms a robust network on its own [8], and the introduction of chitin particles disrupts and interferes this pre-established structure, weakening the gel.

Figure 1c,d show the frequency sweep profiles of the studied oleogels without and with vanillin, respectively, after 15 days of storage. In general, during this storage time, oleogels without chitin were very stable and did not exhibit significant changes, whereas samples containing chitin showed an increase in both elastic (*G*′) and viscous (*G*″) moduli, except for the WV_2.0 oleogel. This last result may be attributed to the fact that so high a chitin concentration could prevent the settlement of the three-dimensional network of the crosslinked vanillin–chitosan structure during storage rather than reinforce it.

On the other hand, the damping factor values (tan δ = *G*″/*G*′) were obtained from frequency sweep assays and correspond to the value at 1 Hz. The values obtained for oleogels without chitin were 0.14 and 0.12 for WOV_0.0 and WV_0.0, respectively, corroborating the marked elastic features of the gels. Damping factor increased with the presence of chitin at low content but decreased as more chitin was added in the oleogels without vanillin, with values ranging from 0.24 for WOV_0.5 to 0.10 for WOV_2.0. The oleogels with vanillin showed a marked elastic character, which was practically not modified by the presence of chitin, with values in the range from 0.09 (WV_2.0 sample) to 0.12 (WV_0.5 sample).

To analyze the structural development of the systems, an exponential equation (Equation (1)) was applied to the *G*′ curves of both fresh vanillin-containing and vanillin-free systems, as well as to the stability data measured after 15 days.

(1) $$G\prime \left(f\right)=A\;f^{n}$$

where *A* is a parameter that corresponds to the gel stiffness at 1 Hz of frequency, *f* is the frequency, and *n* is an exponent that describes the frequency dependence of *G*′.

**Figure 2:**
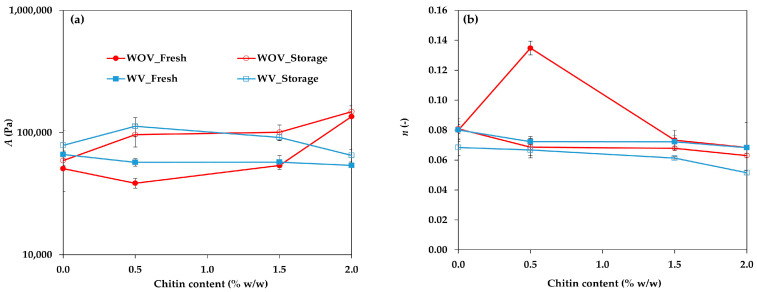
Elastic modulus values at 1 Hz (**a**) and slope values (**b**) of Equation (1) for oleogels formulated with different concentrations of chitin particles.

The obtained values for Equation (1)’s parameters for the studied oleogels are shown in Figure 2a,b. For the vanillin-free samples (red lines), a clear increase in parameter A was observed only at the highest tested chitin content. The oleogels stored for 15 days showed higher values than fresh samples and they increased with chitin content. In contrast, for the oleogels with vanillin (blue line), no significant changes were observed; in fact, it appeared that even small amounts of chitin particles may interfere with the formation of the oleogel network. Additionally, *G*′ values of the vanillin-containing oleogels tended to increase clearly after 15 days, except at the highest chitin content, as was previously commented, which may be attributed to the maturation of the three-dimensional network during storage. These findings led to the conclusion that vanillin enhanced the gel stiffness of oleogels in the absence of chitin or at low chitin concentrations (<1.5% *w*/*w*). Conversely, at higher chitin levels, gel stiffness increased significantly in the absence of vanillin but tended to decrease when vanillin was present. During the storage, the stiffness of vanillin-containing oleogels increased less than that of oleogels formulated without vanillin.

The values of the slope obtained from Equation (1), which describes the elastic modulus dependence with the frequency, are shown in Figure 2b. For the fresh oleogels, with and without vanillin, the slope was practically constant (0.07–0.08), except for the one without vanillin containing 0.5% *w*/*w* chitin particles (WOV_0.5), which showed a value of 0.14. A lower slope indicates a more structured gel. Furthermore, after 15 days, all oleogels showed slightly lower slope values in comparison with their fresh counterparts, meaning that the storage (maturation) period promotes the development of more structured gels.

### 2.2. Oil Binding Capacity

**Figure 3:**
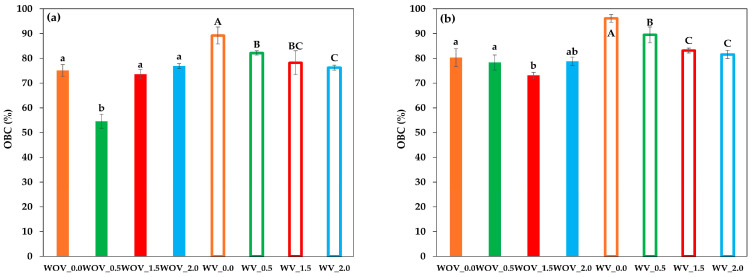
Oil binding capacity (OBC) of chitosan-based oleogels: (**a**) fresh samples and (**b**) samples after 15 days of storage with (WV) and without (WOV) vanillin, and with different chitin particles content. Error bars indicate standard deviations. Different letters denote statistically significant differences (*p* < 0.05) according to Duncan’s multiple range test following ANOVA.

The ability to retain a liquid phase within a solid matrix, known as oil binding capacity (OBC), is attributed to the entrapment of oil within the three-dimensional network formed by structuring agents. This property is critical for the quality and functionality of oleogels, as low OBC values can lead to undesirable visual defects and remarkable nutritional losses in foods. In this study, the effect of chitin particles on chitosan systems, either with or without vanillin, was evaluated in both fresh and oleogels stored for 15 days. The OBC values obtained for the tested gels are shown in Figure 3.

As shown in Figure 3a, fresh oleogels without vanillin exhibited a significant decrease in OBC at the lowest tested chitin content (WOV_0.5: 54.6 ± 2.8%), whereas similar values of the oleogel without chitin were maintained at higher concentrations (WOV_0.0: 75.1 ± 2.4%, WOV_1.5: 73.6 ± 1.8%, and WOV_2.0: 76.9 ± 1.0%), with no significant differences (*p* > 0.05) among these last three samples. Conversely, in oleogels with vanillin, the increase in chitin content decreased OBC values from 89.2 ± 3.5% (WV_0.0) to 76.2 ± 1.0% (WV_2.0). This reduction may be due to the formation of weak zones in the oleogel by chitin particles that facilitate oil leakage.

Previous studies have reported a wide range of OBC values for chitosan-based oleogels (with vanillin and without chitin) using the same range of chitosan content. For instance, Lama et al. [18] studied the effect of air-drying temperature on oleogels, reporting that maximum OBC values (>98%) were obtained at 70 °C, whereas Brito et al. [12] studied the effect of different vanillin–chitosan ratios and found values lower than 68% for freeze-dried oleogels with different vanillin content (in fact, high vanillin content reduced the OBC values). Thus, the OBC values obtained in this study were within the range reported in the literature.

After 15 days of storage (Figure 3b), an overall increase in OBC values was observed for all samples in comparison to fresh samples. Oleogels without vanillin showed OBC values from 73.1 to 80.3%. WOV_1.5 (73.1 ± 1.2%) exhibited a significant decrease compared to WOV_0.0 (80.3 ± 3.6%), WOV_0.5 (78.3 ± 6.1%), and WOV_2.0 (78.8 ± 1.8%) without significant differences between them. Oleogels containing vanillin displayed a similar trend, with OBC values increasing but still showing a chitin concentration-dependent decrease (96.1 ± 1.6% for WV_0.0 down to 81.6 ± 1.7% for WV_2.0).

Regarding the presence of chitin particles, the results presented in this paper contrast with previous reports, such as Silva et al. [21], who demonstrated that oleogel structuring (driven by low-molecular-weight crystalline particles or polymer self-assembly) creates a network that physically traps liquid oil, thereby enhancing the oleogel’s stability and oil retention capacity. The discrepancy observed with our results can be attributed to the relatively large size (20–80 µm) of the chitin particles used in this study, which might hinder the formation of a more efficient oil-trapping network.

Interestingly, OBC values higher than 70% were consistently linked with damping factors lower than 0.2, indicating that oleogels with predominant elastic characters hinder oil release.

### 2.3. Textural Properties

The results of the texture profile analysis of the oleogels are presented in Table 1. The parameters evaluated were hardness, adhesiveness, cohesiveness, and elasticity, measured for both freshly prepared samples and those stored for 15 days. Hardness values of fresh oleogels ranged from 4.7 ± 1.1 to 8.0 ± 1.1 kPa and increased to values between 6.7 ± 0.8 and 10.4 ± 0.3 kPa after 15 days of storage. Adhesiveness and cohesiveness of fresh samples ranged from −0.35 ± 0.09 to −0.98 ± 0.16 N·s and from 0.28 ± 0.02 to 0.37 ± 0.02, respectively, without significant differences being observed after storage. The elasticity of fresh oleogels varied from 38.3 ± 3.1% to 58.0 ± 6.3%, whereas the values obtained for stored samples were between 42.8 ± 4.7 and 60.9 ± 12.8%. Overall, most parameters showed moderate variations during storage, with a general trend to obtain harder oleogels after storage.

In vanillin-free oleogels, the hardness of fresh samples increased significantly from 5.6 ± 0.8 kPa (WOV_0.0) to 8.0 ± 1.0 kPa (WOV_2.0), indicating that higher chitin content reinforces the gel structure. All samples were harder after storage, the highest value being that of WOV_2.0 samples (10.4 ± 0.3 kPa). In contrast, vanillin-containing oleogels displayed a narrower hardness range, from 6.4 ± 1.3 to 7.5 ± 1.3 kPa in fresh samples, regardless of chitin concentration. After storage, a slight but consistent increase was observed for these WV oleogels, indicating structural reinforcement of the samples.

WV oleogels were more adhesive (lower adhesiveness negative values) than their WOV counterparts, independent of chitin content (i.e., −0.59 ± 0.18 and −0.41 ± 0.11 for WV_0.0 and WOV_0.0). Adhesiveness tended to increase with the chitin content in WOV and WV samples, but in a narrow range (the highest value was obtained −0.98 ± 0.16 in WV_1.5). Moreover, it was almost invariant during storage and no significant difference between fresh and stored samples was observed. Cohesiveness did not show significant differences with the presence of vanillin nor with chitin content (*p* < 0.05) for both fresh and stored samples. These results suggest that all tested oleogels showed similar sticking features. The use of vanillin did not change the elasticity value, and no significant differences (*p* > 0.05) were observed between fresh and samples stored for 15 days. With regard to the effect of chitin content on elasticity, the most elastic oleogels were obtained at 1.5% of chitin content, independent of the presence of vanillin (58% and 56% for fresh and stored samples), but with higher chitin content the elasticity dramatically decreased, being obtained in the least elastic samples (45% WV_2.0 and 38% WOV_2.0).

The chitosan- and vanillin-based oleogels studied in this work exhibited textural properties comparable to those reported by Brito et al. [12], who used 0.75% (*w*/*v*) chitosan and 1% vanillin. These authors obtained an oleogel with hardness of 10.2 ± 1.10 N, cohesiveness of 0.65 ± 0.07, and elasticity of 98%. In the present research, WV_0.0 oleogels (fresh oleogels containing 0.65% chitosan and 1.3 vanillin-to-chitosan ratio) showed hardness of 7.5 ± 1.3 kPa, cohesiveness of 0.32 ± 0.02, and elasticity of 48 ± 13%. After storage for 15 days, hardness increased up to 9.5 ± 1.5 kPa. This increase in hardness is consistent with the behavior observed by Brito et al. [12], who reported an increase in hardness after the same storage time (at 35 °C). This change could be associated with the reorganization and compaction processes of the three-dimensional network formed by chitosan and vanillin crosslinking during storage.

### 2.4. Oxidative Stability

**Figure 4:**
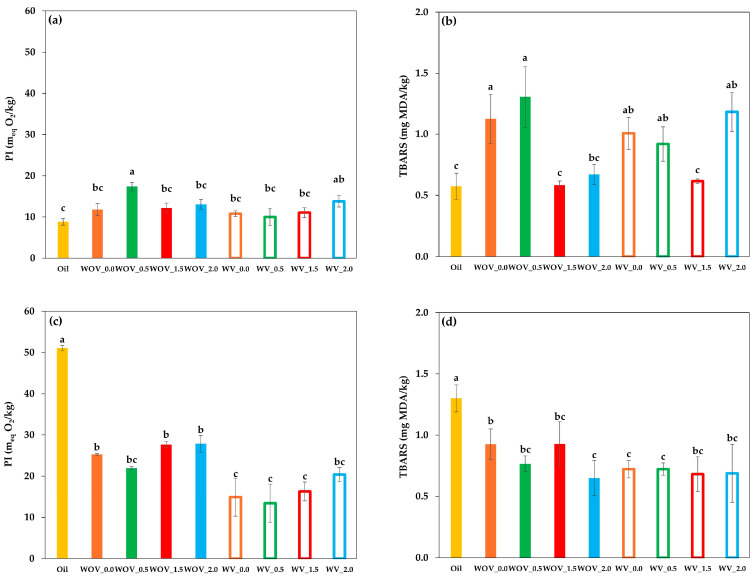
Primary and secondary oxidation in oleogels: (**a**) primary oxidation in fresh oleogels, (**b**) secondary oxidation in fresh oleogels, (**c**) primary oxidation in oleogels stored for 15 days, and (**d**) secondary oxidation in oleogels stored for 15 days. Primary oxidation was assessed by peroxide index (PI), and secondary oxidation by TBARS. Error bars represent standard deviations. Different letters indicate statistically significant differences (*p* < 0.05) according to Duncan’s multiple range test following ANOVA.

Oil oxidation is a key parameter for determining the quality of oleogels; peroxide index (PI), the common method to determine the degree of primary oxidation, ranged from 8.8 meq O_2_ kg^−1^ for pure oil (without processing) to 17.4 meq O_2_ kg^−1^ for the WOV_0.5 oleogel in fresh processed samples (Figure 4a). After 15 days of storage (Figure 4c), the PI values ranged from 13.4 meq O_2_ kg^−1^ for the WV_0.5 oleogel up to 51.1 meq O_2_ kg^−1^ for the pure oil. In general, higher chitin content led to an increase in primary oxidation. This effect may be attributed to the presence of chitin interference with the gel network formation, so that during the maturation of the structure, pores or gaps are formed near the chitin particles (which remain intact), allowing oil to be released through them, thereby increasing its exposure to oxygen and promoting oxidation [21].

Secondary oxidation was assessed by a TBARS assay. As can be seen in Figure 4b, TBARS levels in fresh samples ranged from 0.6 (pure oil) to 1.3 (WOV_0.5). During storage, Figure 4d, the TBARS of oil increased up to 1.3 and oleogels showed values less than 1.0 (with a minimum value of 0.6 for WOV_2.0). No clear trend was observed in relation to chitin concentration, suggesting that the extent of secondary oxidation in oleogels was low and not consistently influenced by the amount of chitin under the conditions tested. In general, these results clearly demonstrate the protective effect of oleogelation against oxidative deterioration. Compared to pure oil, all oleogel systems significantly reduced peroxide formation during storage, confirming the ability of the gel matrix to slow down lipid oxidation and improve oil stability over time.

Evaluating both primary and secondary oxidation of stored oleogels, our results agree with those reported by Farooq et al. [8] who worked with oils structured with natural polymers such as chitosan and vanillin. Oleogels exhibited significantly (*p* < 0.05) lower lipid hydroperoxides and TBARS values as compared with unstructured oils, due to the formation of compact networks that limit oxygen diffusion and enhance oxidative stability. This observation confirms that the gel network’s integrity plays a crucial role in protecting the oil phase from oxidation.

### 2.5. Relationships Among Rheological, Textural, Oil Binding Capacity, and Oxidation Parameters

A correlation matrix was established to identify significant relationships among the properties and parameters obtained from the characterization of oleogels. Correlation values can be seen in Table 2; data above the diagonal corresponds with fresh oleogels, whereas those below the diagonal (in italics) refer to those stored for 15 days.

The most relevant correlations found between parameters obtained from different experimental techniques are briefly discussed below. For fresh oleogels, a strong and significant correlation was observed between hardness and parameter A (r = 0.634; *p* < 0.01) and a moderate and significant correlation with parameter n (r = 0.539; *p* < 0.01). Parameter *A* corresponds to gel stiffness at the reference frequency (1 Hz) and n is an exponent that describes the frequency dependence of the *G*′ modulus measured by rheology. This observation agrees with reports from other authors, who also observed a positive correlation between hardness and *G*′ in oleogels [22,23,24].

On the other hand, hardness also shows a strong and significant positive correlation with OBC (r = 0.675; *p* < 0.01), which is because harder oleogels present a more compact structure and therefore a higher OBC [25,26]. Additionally, a strong and significant positive correlation (r = 0.640; *p* < 0.01) was observed between adhesiveness and the damping factor (tan δ); this may be because tan δ is a parameter that measures the viscoelasticity of the material. This positive correlation suggests that, as the gel behaves more viscously than elastically, it also becomes more adhesive, which is consistent with the behavior of soft or partially fluid viscoelastic materials.

A moderate and significant positive correlation was also observed between peroxide values and TBARS values (r = 0.576; *p* < 0.01), indicating that the progression of primary oxidation in oleogels tends to be associated with an increase in secondary oxidation products. This behavior is consistent with the expected evolution of lipid degradation, where the initially generated hydroperoxides decompose into compounds such as malondialdehyde, detected in the TBARS assay [27,28].

On the other hand, by analyzing the results obtained for oleogels stored for 15 days, a strong and significant negative correlation was observed between OBC and peroxide value (r = −0.657; *p* < 0.01). This indicates that the higher the OBC, the lower the peroxide value obtained, which suggests that a higher oil binding capacity is associated with a lower susceptibility to primary oxidation. This could be explained by the reduction in the exposure of the oil to oxygen thanks to a more efficient oleogel structural network [29,30]. A strong and significant positive correlation between tan δ and adhesiveness (r = 0.796; *p* < 0.01) was also maintained, as it was also reported for fresh oleogels. Additionally, parameter A showed a strong and significant correlation with hardness (r = 0.745; *p* < 0.01), while parameter n correlated significantly with adhesiveness (r = 0.660; *p* < 0.01), and tan δ (r = 0.628; *p* < 0.01). These results indicate that the relationships between rheological parameters and textural properties remain relevant even after 15 days of storage.

## 3. Conclusions

This study presents a first evaluation of the combined influence of vanillin and chitin particles on the properties of chitosan-based oleogels. The findings confirm that vanillin plays a relevant role in promoting the development of structured and functional oleogel matrices. Its inclusion enhanced viscoelastic performance, oil binding capacity, and oxidative stability, attributed to the formation of a compact gel network that restricts oxygen diffusion and protects the oil phase from degradation.

In contrast, the effect of the addition of chitin particles depended on their concentration and on the presence or absence of vanillin. At intermediate levels, chitin decreased the elastic modulus and moderately improved the cohesiveness and elasticity in vanillin-free formulations. However, in vanillin-containing oleogels, chitin appeared to disrupt the chitosan–vanillin network, resulting in slightly weaker mechanical properties, reduced oil retention, and increased susceptibility to primary oxidation during storage (in comparison to oleogels without chitin). These effects can be explained by how chitin acts as a reinforcing agent in oleogels without vanillin due to its ability to interact physically with the chitosan network above certain chitin content, enhancing mechanical strength (with high particle density). At very low chitin content, however, the chitosan gel network is predominantly interrupted by the presence of chitin particles. In systems containing both chitosan and vanillin, the addition of chitin appears to interfere with network formation, likely by partially avoiding the extension of the Schiff base reaction between chitosan and vanillin. This disruption increases with increasing chitin content and leads to a weaker network.

These results suggest that while chitin has potential as a structuring aid when a threshold content is used, its incorporation must be carefully optimized to avoid compromising gel integrity. Future research should explore strategies for improving chitin integration within oleogel matrices such as reducing particle size or applying surface functionalization, while also extending novel formulations to other natural polymers and bioactive compounds to broaden application potential in food, cosmetic, and pharmaceutical fields.

## 4. Materials and Methods

### 4.1. Materials and Reagents

Olive oil (Aceites Abril, S.L., Ourense, Spain) was purchased from a local supermarket, presenting a peroxide concentration of 8.8 ± 1.4 meq O_2_/kg and an acidity percentage of 1%. Medium molecular weight chitosan (300.4 ± 18.3 kg/mol) and a vanillin solution (93% purity) dissolved in 96% ethanol, both obtained from Sigma-Aldrich (St. Louis, MO, USA), were employed as structuring agents. Glacial acetic acid (Merck, Darmstadt, Germany) was used to dissolve the chitosan at 1% *v*/*v*. Chitin particles obtained from commercial chitin (Glentham Life Sciences, Corsham, UK) were prepared following the protocol described by Nikiforidis and Scholten [31], with minor modifications. Chitin (40 g) was bleached with acidified NaOCl at 80 °C for 2 h, filtered, and then treated with 5% KOH solution for 36 h at room temperature to remove residual proteins. After centrifugation, the resulting pellet was hydrolyzed in boiling 3 N HCl for 90 min, diluted with distilled water, centrifuged again, and dialyzed in distilled water for 12 h. Finally, the dispersion was adjusted to pH 3, frozen at −32 °C, and freeze-dried at −60 °C and 0.011 mbar for 5 days to obtain dry chitin particle powder. The resulting range of chitin particles size was from 20 to 80 µm (mean diameter of 29.6 ± 3.8 µm), measured by optical microscopy (Zeiss Axioskop 40, Jena, Germany) equipped with a digital camera (Nikon D70, Tokyo, Japan).

### 4.2. Emulsion and Oleogel Preparation

The emulsions were prepared following the protocol described by Lama et al. [18], with some modifications. A total of 50 g of oil-in-water emulsions (50% *w*/*w*) were prepared for each experiment. The aqueous phases consisted of a solution of chitosan alone (WOV) and with vanillin (WV) and chitin particles. The chitosan concentration used in the emulsions was 0.65% *w*/*w*. The vanillin was added at a constant vanillin-to-chitosan ratio of 1.3 [18]. The following content of chitin particles were used: 0.0, 0.5, 1.5, and 2.0% *w*/*w* based on the mass of emulsion.

Olive oil was added to the chitosan solution at ~9 mL/min using a burette while stirring with an orbital shaker (P-Selecta Rotaterm, Barcelona, Spain) at 150 rpm. The mixture was then homogenized using a high-shear disperser (Ultraturrax T-25 Basic, IKA-WERK, Staufen, Germany) at 9500 rpm for 3.5 min, after which the vanillin solution was added to complete a total homogenization time of 4 min. The emulsion was further homogenized for an additional 4 min, gently stirred at 400 rpm for 2 h at room temperature, and then allowed to rest for 24 h to ensure completion of the chitosan–vanillin reaction.

The emulsions were then dried in Petri dishes of 1.5 mm thickness into a forced convective air dryer (Angelantoni Challenge 250, Massa Martana, Italy) at 70 °C, maintaining a constant air velocity of 2 m/s and low relative humidity (10%), to remove moisture and residual ethanol. Drying continued until a dried solid was formed, with final moisture content below 1%. The presence of vanillin influenced the drying time, requiring approximately 95 min for the emulsion without vanillin and 120 min when vanillin was added. Drying time also increased with chitin concentration. For instance, at the highest chitin concentration tested (2.0%), elapsed drying time was approximately 300 min, whereas at 1.5% chitin, it was 200 min.

Dried samples were placed in beakers and homogenized at 9500 rpm for a time adjusted according to sample weight, using a ratio of 2 min per 100 g of sample. The resulting oleogels were stored at 4 °C for 48 h prior to characterization (fresh samples). Finally, oleogels were stored at room temperature for 15 days and then analyzed (stored samples). All experiments were performed in triplicate to ensure reproducibility.

### 4.3. Oleogel Characterization

#### 4.3.1. Rheological Characterization

The rheological properties of the oleogels were analyzed using a stress-controlled rheometer (Anton Paar MCR 301, Anton Paar, Graz, Austria) equipped with a parallel plate geometry (50 mm diameter, 1.0 mm gap) at 25 °C. The linear viscoelastic region (LVR) was first determined via an amplitude sweep ranging from 0.01 to 1000% strain at a fixed frequency of 1 Hz. Subsequently, a frequency sweep from 0.1 to 100 Hz was performed at 1% strain to assess the viscoelastic properties of the samples. Temperature control was achieved using a Peltier system with a precision of ±0.01 °C. All measurements were performed in triplicate.

#### 4.3.2. Oil Binding Capacity

The oil binding capacity (OBC) of the oleogels was evaluated following the methodology described by Morales et al. [32], with slight modifications. Oleogel samples (1 g) were placed into pre-weighed Eppendorf tubes and centrifuged at 9170× *g* for 25 min at 20 °C using a centrifuge (HWLAB, HW12, Shiley, NW, USA). The released oil (supernatant) was carefully removed with a Pasteur pipette, and the tubes were weighed again. The OBC (%) was calculated using the following equation (Equation (2)):

(2) $$\mathrm{OBC}\;\left(\%\right)=\left(\frac{m_{2}-m}{m_{1}-m}\right)\cdot 100$$

where *m* (g) is the weight of the empty Eppendorf tube, and *m*_1_ (g) and *m*_2_ (g) are the weight of the Eppendorf tube with oleogel before and after centrifugation.

#### 4.3.3. Textural Properties

Oleogel samples with an average diameter of 19 mm and height of 8.5 mm were subjected to compression testing using a texture analyzer (TA.XT Plus, Stable Micro Systems, Surrey, UK) equipped with a cylindrical probe (25 mm diameter, SMS P/25). Six replicates were analyzed for each oleogel formulation. Tests were conducted at 50% compression, with test conditions based on the methodology described by Farooq et al. [8]. An initial trigger force of 0.1 N was applied, and the pre-test, test, and post-test speeds were set at 2, 1, and 2 mm/s, respectively. From these tests, the maximum hardness (kPa), adhesiveness (N s), cohesiveness (–), and elasticity (%) were determined.

#### 4.3.4. Oxidative Stability

The oxidation degree of oleogels was assessed by extracting the oil phase through centrifugation at 9576× *g* for 20 min. Primary oxidation was evaluated by measuring the peroxide index (PI), whereas the secondary oxidation degree was assessed via a TBARS assay. The PI determination followed a modified version of the American Oil Chemists’ Society method CD-8b90 [33]. In brief, 0.3–0.5 g of oil extracted from oleogel samples was dissolved in chloroform, followed by sequential addition of glacial acetic acid, water, potassium iodide, and potato starch as visual indicators. The peroxide value was calculated by titrating with sodium thiosulfate until the blue color disappeared, indicating the endpoint. Results were expressed as milliequivalents of active oxygen per kilogram of oil (meq O_2_ kg^−1^).

The extent of secondary lipid oxidation in oleogels was assessed by using the thiobarbituric acid reactive substances (TBARS) assay, following the protocol reported by Zhao et al. [34]. Briefly, 0.5 g of each oleogel sample was mixed with a reagent solution consisting of 2.0% (*w*/*w*) hydrochloric acid, 0.375% (*w*/*w*) thiobarbituric acid (TBA), and 15% (*w*/*w*) trichloroacetic acid. The mixtures were incubated in a water bath at 95 °C for 15 min to facilitate the reaction and further cooled to room temperature for 15 min and filtered (0.45 µm). The absorbance of the resulting supernatant was measured at 532 nm using a UV–Vis spectrophotometer. TBARS concentrations were quantified using a standard calibration curve obtained with 1,1,3,3-tetraethoxypropane as standard.

### 4.4. Statistical Analysis

The effect of chitin particle concentrations and the presence of vanillin on oil binding capacity, textural and rheological parameters, and oxidation values were examined using an analysis of variance (ANOVA). The least squares mean (LSM) were separated into statistically significant different groups applying Duncan’s post hoc test. All statistical tests of LSM were performed for a significance level α < 0.05. Correlations between variables (*p* < 0.05 and *p* < 0.01) were determined by correlation analyses using Pearson’s linear correlation coefficient. All statistical analyses were performed using the software IBM SPSS Statistics 29.0 (IBM Corp, Armonk, NY, USA).

## Figures and Tables

**Table 1:** Texture of oleogels with (WV) and without (WOV) vanillin at several chitin contents, evaluated in fresh samples and after 15 days of storage. Results are expressed as mean ± standard deviation. Different letters indicate statistically significant differences between samples (*p* < 0.05); lowercase letters (a–c) refer to fresh samples, and uppercase letters (A–D) refer to stored samples.

Oleogel	Storage (Days)	Hardness (kPa)	Adhesiveness (N s)	Cohesiveness (-)	Elasticity (%)
WOV_0.0	0	5.6 ± 0.8 ^bc^	−0.41 ± 0.11 ^a^	0.34 ± 0.03 ^abc^	50.1 ± 11.1 ^ab^
WOV_0.5		4.7 ± 1.2 ^c^	−0.35 ± 0.09 ^a^	0.36 ± 0.04 ^ab^	50.0 ± 8.6 ^ab^
WOV_1.5		5.7 ± 0.6 ^bc^	−0.65 ± 0.09 ^ab^	0.37 ± 0.02 ^a^	58.0 ± 6.3 ^a^
WOV_2.0		8.0 ± 1.1 ^a^	−0.61 ± 0.17 ^ab^	0.29 ± 0.02 ^c^	38.3 ± 3.1 ^b^
WV_0.0		7.5 ± 1.3 ^ab^	−0.59 ± 0.18 ^ab^	0.32 ± 0.02 ^bc^	47.8 ± 13.1 ^ab^
WV_0.5		7.4 ± 1.2 ^ab^	−0.54 ± 0.28 ^ab^	0.32 ± 0.04 ^abc^	49.4 ± 10.8 ^ab^
WV_1.5		7.1 ± 0.5 ^ab^	−0.98 ± 0.16 ^c^	0.37 ± 0.02 ^a^	57.8 ± 9.8 ^a^
WV_2.0		6.4 ± 1.3 ^abc^	−0.80 ± 0.12 ^bc^	0.33 ± 0.03 ^abc^	45.3 ± 6.1 ^ab^
WOV_0.0	15	6.7 ± 0.8 ^C^	−0.34 ± 0.12 ^A^	0.32 ± 0.04 ^AB^	46.9 ± 9.4 ^A^
WOV_0.5		7.2 ± 1.5 ^C^	−0.45 ± 0.21 ^AB^	0.31 ± 0.04 ^AB^	60.9 ± 12.8 ^A^
WOV_1.5		7.9 ± 1.4 ^BC^	−0.63 ± 0.05 ^ABC^	0.38 ± 0.08 ^A^	56.4 ± 15.7 ^A^
WOV_2.0		10.4 ± 0.3 ^A^	−0.71 ± 0.09 ^BCD^	0.27 ± 0.01 ^B^	42.8 ± 4.7 ^A^
WV_0.0		9.5 ± 1.5 ^AB^	−0.49 ± 0.17 ^AB^	0.30 ± 0.03 ^AB^	44.7 ± 7.3 ^A^
WV_0.5		9.5 ± 1.5 ^AB^	−0.52 ± 0.25 ^AB^	0.30 ± 0.05 ^AB^	45.5 ± 8.3 ^A^
WV_1.5		7.8 ± 0.9 ^BC^	−0.99 ± 0.19 ^D^	0.38 ± 0.03 ^A^	57.3 ± 9.5 ^A^
WV_2.0		8.6 ± 0.3 ^ABC^	−0.91 ± 0.12 ^ACD^	0.32 ± 0.02 ^AB^	46.9 ± 5.0 ^A^

**Table 2:** Pearson correlation coefficients between relevant parameters. Values above the diagonal correspond to fresh oleogels; values below the diagonal (in italics) correspond to oleogels stored for 15 days. Bold values indicate significant correlations (** *p* < 0.01, * *p* < 0.05).

	Hardness	Adhesiveness	Cohesiveness	Elasticity	PI	TBARs	OBC	tan δ	A	n
Hardness (kPa)	-	0.034	−0.032	0.223	−0.337	−0.223	0.675 **	−0.400	0.634 **	−0.539 **
Adhesiveness (N s)	*0.151*	-	0.196	0.275	0.390	0.510 *	−0.280	0.640 **	0.013	0.507 *
Cohesiveness	*0.070*	*0.035*	-	0.909 **	0.259	0.068	−0.258	0.333	−0.408 *	0.231
Elasticity (%)	*0.122*	*0.286*	0.831 **	-	0.043	−0.021	0.016	0.201	−0.223	0.054
PI (meq O_2_ kg^−1^)	*−0.059*	*0.175*	*0.076*	*0.143*	-	0.576 **	−0.702 **	0.666 **	−0.072	0.687 **
TBARs (mg MDA/kg)	*−0.199*	0.451 *	*0.396*	*0.352*	0.454 *	-	−0.266	0.577 **	−0.368	0.516 **
OBC (%)	0.412 *	*0.193*	*−0.159*	*−0.175*	−0.657 **	*−0.106*	-	−0.725 **	0.303	−0.776 **
tan δ (-)	*−0.172*	0.796 **	*0.152*	*0.400*	*0.000*	*0.395*	*0.142*	-	−0.343	0.924 **
A (Pa)	0.745 **	*0.246*	*0.220*	*0.378*	*0.251*	*−0.048*	*−0.022*	*0.027*	-	−0.388
n (-)	*−0.316*	0.660 **	*−0.104*	*−0.045*	*0.213*	*0.349*	*−0.054*	0.628 **	*−0.146*	-

## Data Availability

The raw data supporting the conclusions of this article will be made available by the authors on request.
